# Incidental Abdominal Wall Mass Diagnosed As Endometriosis: A Rare Finding in an Increasingly Common Pathology

**DOI:** 10.7759/cureus.80286

**Published:** 2025-03-09

**Authors:** Taylor Nicely, Lauren Lim, Avarie Willette, Julia R Legiec, Hussain Rawiji

**Affiliations:** 1 Obstetrics and Gynecology, Lake Erie College of Osteopathic Medicine, Bradenton, USA; 2 College of Medicine, Lake Erie College of Osteopathic Medicine, Bradenton, USA; 3 Pediatrics and Obstetrics, AdventHealth Fish Memorial, Orange City, USA

**Keywords:** abdominal-wall endometriosis, chronic abdominal pain, fibroid uterus, heavy vaginal bleeding, hormone therapy, perimenopause, rare case report

## Abstract

Endometriosis is becoming a well-discussed topic in the medical field of women's health, but rare and uncommon pathologic cases such as abdominal wall endometriosis are often overlooked in a patient’s differential diagnoses. This is likely due to the need for greater awareness of its diverse clinical presentations, its impact on patient well-being, and the limitations in clinical suspicion, imaging accuracy, and treatment approaches. Although abdominal wall endometriosis is increasingly diagnosed, healthcare providers remain hesitant to prioritize it - along with endometriosis in general - as a primary diagnosis. In this paper, we discuss a case of a 51-year-old perimenopausal G2P2 female who presented to the emergency department with chief complaint of heavy vaginal bleeding for the past week. Physical exam revealed mild tenderness in the suprapubic area with notable diffuse masses, but was otherwise normal with no mass felt on abdominal palpation. Initial lab results at admission showed hemoglobin at a critical value of 5.4, for which the patient was immediately started on red blood cell transfusion. A transvaginal pelvic ultrasound resulted in fibroid uterus, normal sonographic appearance of endometrial complex, and nonvisualization of either ovary. Given the patient’s extensive reproductive history and the need to rule out common causes of abnormal uterine bleeding, such as fibroids, adenomyosis, endometrial hyperplasia, and malignancy, the decision was made to proceed with a supracervical subtotal hysterectomy with bilateral salpingo-oophorectomy. During the procedure, an incidental abdominal mass was discovered and partially resected to allow for further investigation. After being reviewed by pathology, a rare finding was revealed, that is, abdominal wall endometriosis. The emphasis of this case is to describe the rarity of abdominal wall endometriosis, the clinical significance of early recognition by including abdominal wall endometriosis in the differential list, and to explore the different diagnostic and treatment modalities available, and all with the goal of providing further awareness for clinicians to consider abdominal wall endometriosis as a diagnosis in women premenarche, perimenopause, and postmenopause.

## Introduction

Endometriosis is defined as endometrial stroma and glands found outside of the uterine cavity and can present in multiple locations. The most common of these locations is within the pelvis, but endometriosis has been found in extra-pelvic sites: the abdominal cavity, bowels, and lungs [1]. Deep lesions can also be found in the pouch of Douglas, posterior vaginal fornix, and uterosacral and cardinal ligaments [1]. Endometriosis is not considered cancerous or life-threatening but may commonly present with a sequela of uncomfortable symptoms: dyspareunia, dysmenorrhea, dyschezia, and heavy menstrual bleeding. In addition, endometriosis may be a cause of infertility, unbearable chronic pain, and bowel and bladder dysfunction that prompts women to seek treatment [1]. It is important to note that it is possible for women with endometriosis to be asymptomatic, and findings are often found incidentally during a surgery indicated for another medical condition [1]. This patient presented with irregular bleeding and lacked any complaints of typical endometriosis. This represents an example of a patient who would benefit greatly from a widened spectrum of differential diagnoses.

Endometriosis is an estrogen-dependent inflammatory disease that is believed to be caused by retrograde menstruation. In this process, endometrial cells travel backward through the fallopian tubes and the pelvic cavity and implant in these locations during menstruation. This leads to the implantation of ectopic endometrial tissue and the symptoms experienced by women with endometriosis [2]. Because endometriosis is an estrogen-dependent process, the finding of abdominal wall endometriosis in our patient is considered to be a significant finding, considering she is at the age window of approaching menopause, and physiologically, there is typically weaker cyclic hormonal stimulation as women age and estrogen levels decline [1]. The most studied risk factor for abdominal wall endometriosis is a history of cesarean section or abdominal wall surgery, as endometrial tissue can leak out of the uterus and implant in the abdominal cavity during the procedure [3]. More specifically, surgery disrupts tissue barriers, which can foster a more favorable environment for endometrial tissue to grow. The resulting inflammation and potential adhesions may lead to angiogenesis as well, supporting the growth of the mass [3]. Given that the patient had a significant past medical history of two cesarean sections, it is possible that her past abdominal surgical history increased her chances of developing abdominal wall endometriosis. However, given the patient’s lack of cyclic pain as would be seen with abdominal wall endometriosis, the differential diagnoses of hernia, abscess, and tumor aligned more with her clinical presentation.

The American Society for Reproductive Medicine classifies endometriosis into four different stages based on the severity of the disease. The American Society for Reproductive Medicine's classification system is the most widely used today. The stage is determined by a cumulative score reflecting lesion locations and adhesion severity: Stage 1 (minimal) corresponds to 1-5 points, Stage 2 (mild) to 6-15 points, Stage 3 (moderate) to 16-40 points, and Stage 4 (severe) to more than 40 points. Notably, pain and infertility are not considered in disease staging [3].

Endometriosis roughly affects 10% of reproductive-aged women, amounting to an estimated 190 million individuals worldwide. Most cases occur in women aged 25-35 years, though rare cases have been reported in premenarchal, perimenopausal, and postmenopausal individuals [3]. A UK study done in 2024 by the All-Party Parliamentary Group found that 58% of women diagnosed with endometriosis had made multiple visits to their general practitioner before receiving an official diagnosis of endometriosis [3]. The study also found that the average time-to-diagnosis was eight years, and the reason for the delay in diagnosis was most likely due to similar symptoms occurring in both endometriosis and primary dysmenorrhea, making it difficult for patients and physicians alike to realize they have signs and symptoms of endometriosis [3].

Physical examination findings for endometriosis may vary between patients depending on the amount and location of ectopic endometrial tissue but may reveal focal tenderness with the vaginal examination, nodular masses in the adnexa, and an immobile cervix or uterus. A physical examination may reveal no findings, as was the case in this patient, but this should not exclude the diagnosis [1]. In terms of laboratory markers, CA 125 has been seen to be elevated in some cases of endometriosis, but is not routinely measured in these patients since it can be elevated from other causes such as ovarian cancer. Imaging modalities, including transvaginal ultrasound and MRI, can be used to identify endometriosis lesions and help guide different surgical approaches, but the gold standard is laparoscopic evaluation [1]. Imaging findings may include endometriomas and bladder nodules on transvaginal ultrasound, while chest CT can identify thoracic endometriosis. Abdominal wall endometriosis may present as a solid, hypoechoic mass with significant infiltration of nearby structures on ultrasound [1].

Although the surgical diagnosis of endometriosis is the current gold standard, presumptive diagnosis of endometriosis based on physical exam, imaging findings, and symptoms alone has gained favor [1]. This is due to being less invasive in comparison to surgery, as well as more affordable options of initial treatment, including nonsteroidal anti-inflammatory drugs, gonadotropin-releasing hormone agonists and antagonists, estrogen-progestin, and progestin hormonal contraceptives being available for initial intervention [1]. Although these medications may be effective in eradicating symptoms of endometriosis, response to these treatments should not be used to confirm or exclude endometriosis. Surgical evaluation remains the gold standard to confirm the condition [1].

Surgical diagnosis with tissue biopsy is the favorable choice in individuals who do not respond well to the initial therapies listed above, as well as those who want a definitive diagnosis. Surgical diagnosis can also treat the condition while confirming the diagnosis at the same time, making it a favorable option in some cases [1]. Instances that call for surgical diagnosis over presumptive include severe pain that significantly limits function, pain that persists despite medical intervention, and severe symptomatic anatomic lesions such as ovarian cysts or bladder lesions [1]. Upon surgical evaluation, white opacifications or blue-brown lesions, known as “powder-burn” lesions, may be seen [1].

In patients where these lesions are seen but histology is negative, they are treated for endometriosis due to the possibility of inadequate tissue sampling. In situations where there are no visual lesions and negative histology, endometriosis can be excluded as a diagnosis. The early identification, diagnosis, and treatment of endometriosis may substantially improve the quality of life in many women, as well as preserve fertility, making it an essential topic to discuss in healthcare [1].

## Case presentation

The patient was a 51-year-old perimenopausal G2P2 female who presented to the emergency department with heavy vaginal bleeding for the past week. The patient stated that the bleeding had been far more intense than before and described changing one heavy tampon and overnight pad per hour and passing clots the size of baseballs. She admitted to experiencing some mild cramping and feeling really shaky but refused any pain medications. She admitted to a positive family history of endometriosis. Her past medical history included heavy menstrual periods, fibroids, ovarian cysts, two cesarean sections, the removal of precancerous cervical cells in her teens, and periurethral cyst removal in her teens. She reports that her last gynecological exam was seven years ago, and she has not followed up for further checkups since then. She states the periods have mostly been normal, lasting about four days, and she gets them every 28 days except the most recent. The physical exam was unremarkable except for mild tenderness in the suprapubic area with notable diffuse masses assumed to be uterine fibroids. No notable mass was felt on the physical exam of the abdomen.

In the emergency department, the patient was given a 250 mL infusion of sodium chloride 0.9% and a 15 mg intravenous injection of ketorolac (Toradol). Laboratory tests, a transvaginal ultrasound, and an electrocardiogram were also ordered. The complete blood count showed abnormal results, including anemia with neutrophilia, elevated prothrombin time, and abnormal morphology positive for anisocytosis, hypochromia, and microcytes, with adequate platelets. The patient’s symptoms of shakiness and mild cramping are consistent with severe anemia and ongoing blood loss. Despite adaptation to chronic anemia, the hemoglobin of 5.4 g/dL was notably low, necessitating immediate red blood cell transfusion to prevent further hemodynamic instability. The microcytic, hypochromic pattern strongly suggests iron deficiency anemia, requiring iron supplementation and further evaluation of the underlying cause of the bleeding. The patient’s complete blood count levels are shown in Table 1.

**Table 1:** Patient lab values upon arrival at the emergency department. WBC: white blood cell; RBC: red blood cell; MCV: mean corpuscular volume; MCH: mean corpuscular hemoglobin; MCHC: mean corpuscular hemoglobin concentration; RDW: red cell distribution width; MPV: mean platelet volume

CBC	Reference	Patient values on admission
WBC	4.80-10.80 x 10^3^/uL	8.5 x 10^3^/uL
RBC	4.20-5.40 x 10^6^/uL	2.66 x 10^6^/uL
Hemoglobin	12.0-16.0 g/dL	5.4 g/dL
Hematocrit	37.0-47.0%	18.8%
MCV	80.0-99.0 fL	70.7 fL
MCH	27.0-33.0 pg	20.3 pg
MCHC	27.0-33.0 pg	28.7 pg
RDW	11.9-17.7%	18.1%
Platelet count	11.9-17.7%	251%
MPV	7.4-10.4 fL	9.8 fL
Neutrophils	45.0-70.0%	72.2%
Lymphocytes	45.0-70.0%	19.4%
Monocytes	4.2-12.4%	6.1%
Eosinophils	0.0-6.0%	1.3%
Basophils	0.0-2.3%	0.5%
Neutrophils absolute	1.80-7.20 x 10^3^/uL	6.14 x 10^3^/uL
Lymphocytes absolute	1.10-4.20 x 10^3^/uL	1.65 x 10^3^/uL
Monocytes absolute	0.00-0.70 x 10^3^/uL	0.52 x 10^3^/uL
Eosinophils absolute	0.00-0.50 x 10^3^/uL	0.11 x 10^3^/uL
Basophils absolute	0.00-0.22 x 10^3^/uL	0.04 x 10^3^/uL

**Figure 1:**
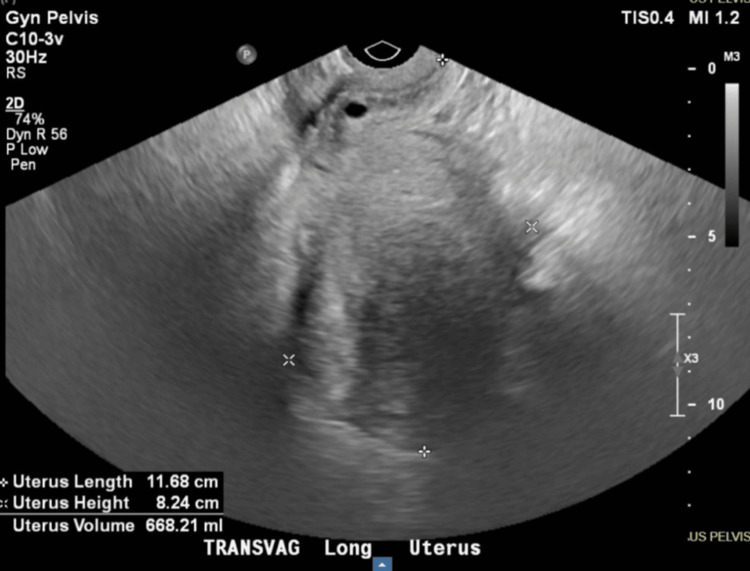
Non-obstetric transvaginal ultrasound showing an enlarged uterus.

**Figure 2:**
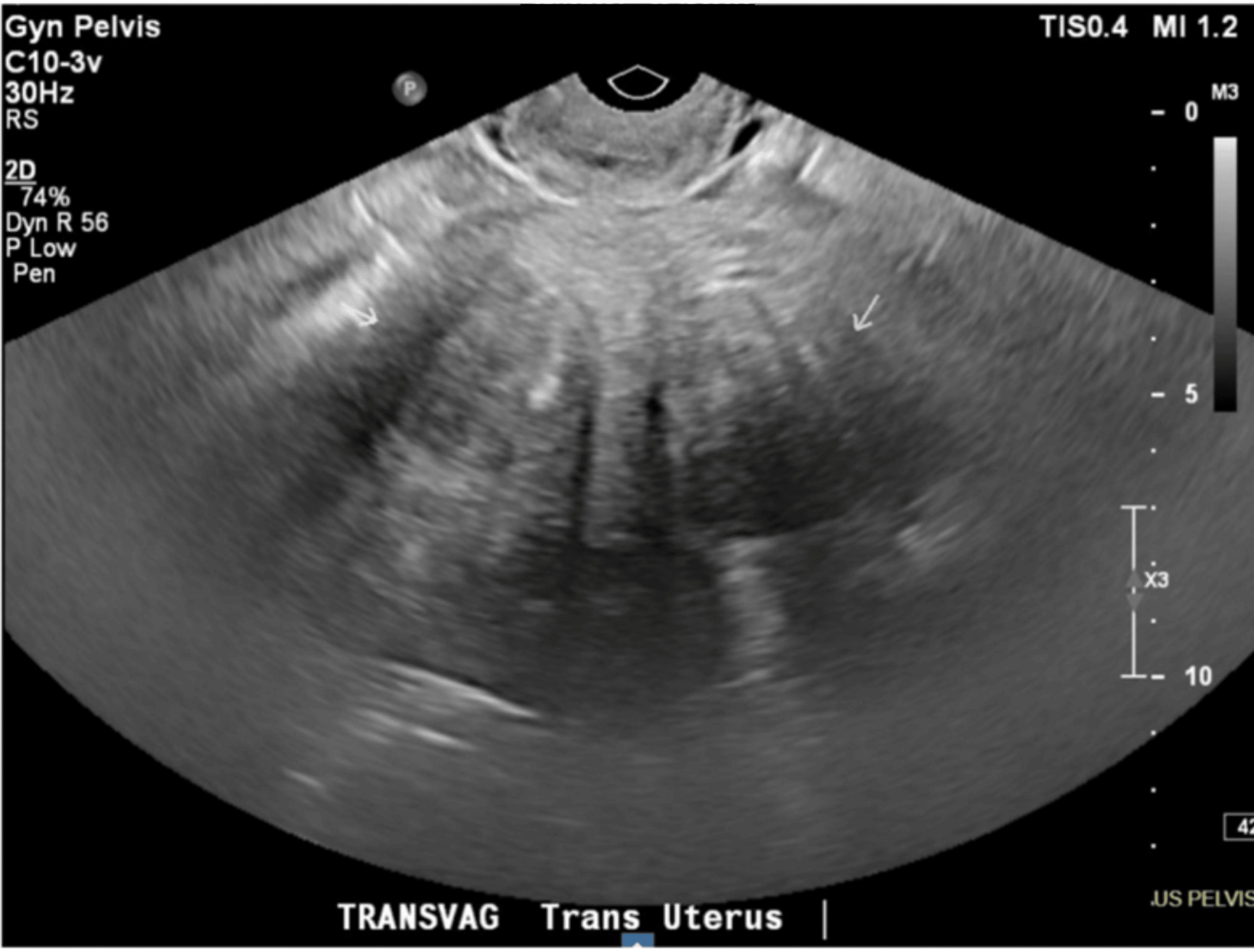
Non-obstetric transvaginal ultrasound showing multiple (at least two) heterogeneously isoechoic shadowing intramural fibroids, indicated by arrows, as follows: left uterine body measuring 3.9 × 5.2 × 4.7 cm; right uterine body measuring 4.2 × 3.8 × 4.0 cm.

A transvaginal pelvic ultrasound revealed a fibroid uterus, normal sonographic appearance of endometrial complex, and non-visualization of either ovary. The patient’s heavy vaginal bleeding and critically low hemoglobin indicate that symptomatic uterine fibroids are the likely source of blood loss, as confirmed by ultrasound findings of a fibroid uterus. Due to the severity of anemia and persistent symptoms, a supracervical subtotal hysterectomy with bilateral salpingo-oophorectomy or uterine artery embolization was suggested to control the bleeding, prevent further anemia, and relieve fibroid-related symptoms. The patient was informed about the longer period of time that the embolization may take to shrink the fibroids, and the patient opted for a hysterectomy. The patient voiced concern about leaving the cervix behind if possible but was okay with having it removed if abnormal. Figures 1-2 show the transvaginal pelvic ultrasound measurements and findings showcasing multiple intramural fibroids.

**Figure 3:**
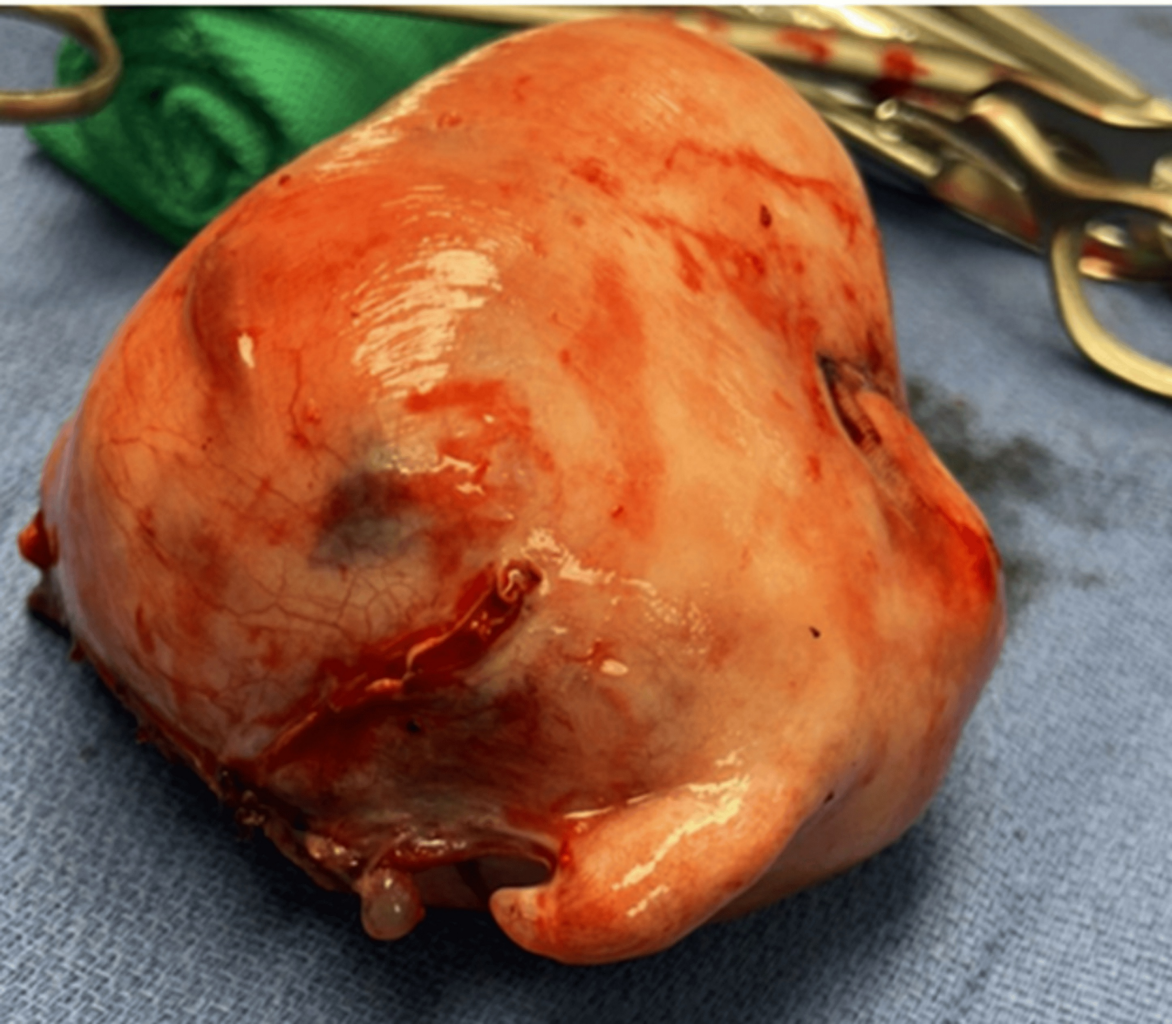
The patient’s isolated, enlarged uterus containing intramural fibroids.

Surgery was performed on day 2 of admission and was labeled an emergency hysterectomy. At the beginning of surgery, it was noted that she had a very hard section of her abdomen from the suprapubic area to one-third of the way to the umbilicus, which was described as a potential protruding fibroid that was in the way of the surgical field once the patient was opened up. Additional tissue had to be removed from the large mass to gain access to the operating site. Two large fibroids were observed, and the bowel was stuck to the uterus, ovarian tubes, and ovaries on the left side, which took significant time to separate carefully. Once the uterus was isolated, each fibroid was injected with a dilute solution of lidocaine with epinephrine to cause blanching and decrease blood loss. Abnormal cells discovered on the cervix led to cervical removal. The vagina was supported with uterus sacral ligaments on each side. Figure 3 depicts a uterus that was removed with fibroids present internally.

Once the hysterectomy was completed, the large mass that remained measured 3 cm deep, 5 cm wide, and 5-7 cm caudad. A very small portion of this mass was removed for tissue biopsy, and intraoperative consultation from the general surgeon was requested. It was decided to wait for the biopsy results and have the patient follow up in the office. The patient was closed with difficulty due to the tissue having gone partly into the fascia. The patient was made aware of this mass and advised to follow up with the general surgeon. ​​The abdominal mass biopsy specimen consisted of fatty tissue consistent with omentum. Further sectioning revealed an ill-defined area of indurated, white, granular tissue with areas of hemorrhage or cysts, grossly consistent with endometriosis.

## Discussion

Abdominal wall endometriosis is a rare presentation of endometriosis. It typically occurs in women with a history of abdominal or pelvic surgeries such as cesarean sections. The pathophysiology of abdominal wall endometriosis is thought to be a result of the mechanical implantation of endometrial cells during surgical procedures, where they adhere to the abdominal wall and proliferate in response to hormonal stimuli [4]. This case is notable due to the incidental finding of abdominal wall endometriosis in a perimenopausal patient without classical cyclic pelvic pain, broadening the differential diagnosis possibilities for abdominal wall masses. Table 2 outlines the American Society for Reproductive Medicine classifications of endometriosis based on the severity of the disease.

**Table 2:** The American Society for Reproductive Medicine (ASRM) classification of endometriosis stages.

Stage	Severity	Point range	Description
Stage 1	Minimal	1-5	Few superficial lesions, minimal adhesions
Stage 2	Mild	6-15	More lesions, deeper implants, mild adhesions
Stage 3	Moderate	16-40	Many deep implants, cysts on ovaries, adhesions
Stage 4	Severe	>40	Extensive lesions, large cysts, dense adhesions

The diagnosis of abdominal wall endometriosis is challenging due to its non-specific symptoms, such as localized pain, swelling, or mass formation in the abdominal wall. In many cases, abdominal wall endometriosis is misdiagnosed as a hernia, lipoma, or neoplasm since the symptoms often overlap [4]. In this patient, the incidental discovery of an abdominal mass during a hysterectomy for abnormal uterine bleeding highlights the importance of considering abdominal wall endometriosis in the differential diagnosis of vaginal bleeding as well as abdominal or pelvic masses, especially in patients outside the typical reproductive age group. One such case describes a 44-year-old premenopausal, nulliparous woman who presented with ascites and a large abdominal mass arising from a lower midline laparotomy scar, which was discovered to be endometrial tissue [5]. Transvaginal ultrasound is the first line test for diagnosing endometriosis, but when a patient presents with an extensive medical history, other testing should also be considered to possibly allow for better viewing of abdominal wall endometriosis: axial T1-weighted fat-saturated spin-echo MRI after contrast injection, contrast-enhanced CT scan, and power Doppler sonogram [6].

Endometriosis is predominantly seen in reproductive-aged women, affecting approximately 10% of this population [3]. However, endometriosis can still be found in premenopausal and, more rarely, perimenopausal and postmenopausal women, as in this case. Perimenopausal and postmenopausal endometriosis is uncommon due to the decline in circulating estrogen, however, hormone replacement therapy or residual ectopic endometrial tissue may continue to stimulate endometriosis lesions. Our patient had a significant gynecological history, including multiple surgeries, which likely contributed to the implantation and subsequent growth of endometrial tissue in her abdominal wall, despite being postmenopausal. Surgical procedures, particularly those involving the uterus, can facilitate the migration and implantation of endometrial cells in extra-pelvic locations, such as the abdominal wall, where they can proliferate even in the absence of menstruation [3].

The patient’s clinical presentation of heavy vaginal bleeding and severe anemia was not initially suggestive of endometriosis. Her surgical history and physical findings, along with imaging studies, indicated a need for a hysterectomy, which ultimately revealed the abdominal wall mass. The mass was later confirmed via pathology to be endometriosis, highlighting the need for a comprehensive evaluation of incidental findings during surgery to ensure accurate diagnosis and optimal patient management.

Management of abdominal wall endometriosis typically involves surgical excision, which provides both diagnostic value and symptom relief. Medical management with hormonal therapies, including gonadotropin-releasing hormone (GnRH) agonists, oral contraceptives, and progestins, is often employed in pelvic endometriosis but may not be effective for abdominal wall endometriosis. In addition, GnRH agonists have FDA approval for a short interval, from 6 to 24 months [7]. Therefore, while these treatments are successful in reducing pain associated with endometriosis, they are not considered a long-term therapy option [7]. The patient was initially offered hormone replacement therapy and oral contraceptives as treatment modalities available, but the patient declined the use of any medical management due to personal preference. In this case, the patient underwent surgical excision, which was necessary due to the size and involvement of the mass in the surgical field. Due to the mass’s infiltration into the surrounding fascia, complete removal was challenging. However, incomplete excision can lead to recurrence. Long-term follow-up is essential to monitor for recurrence and to address any lingering symptoms.

## Conclusions

This case is unusual as it challenges the belief that endometriosis is limited to reproductive-aged females and presents a unique manifestation of the disease in the abdominal wall. It highlights the variety of presentations that may occur in endometriosis, as well as the importance of early symptom recognition, diagnosis, treatment, and patient education. Endometriosis should remain on the differential, even in perimenopausal and postmenopausal patients. The delay in diagnosis may be attributed to the overlap of symptoms with primary dysmenorrhea, making differentiation difficult based on symptoms alone. Many women with endometriosis experience chronic pain, heavy menstruation, dyspareunia, and bowel or bladder dysfunction. However, some patients do not present with these cyclical episodes, further contributing to the diagnostic challenge.

The presence of a large abdominal wall mass without significant pain underscores the need for clinicians to consider abdominal wall endometriosis in perimenopausal, postmenopausal, and atypical cases. Patients could benefit significantly from early pharmacologic interventions, advanced imaging modalities, and surgical diagnosis with subsequent treatment. A higher suspicion for endometriosis should be maintained in individuals with a history of gynecologic surgeries, such as cesarean sections, even in the absence of a "classic" presentation. Ultrasound should be utilized for evaluation in these cases. Many healthcare providers may not fully recognize how distressing this condition is for patients, which can contribute to the normalization of symptoms and delays in diagnosis. Consequently, it is imperative that both healthcare providers and patients expand their knowledge of the condition, including the broader range of age groups in which abdominal wall endometriosis can occur, as prompt intervention can significantly improve quality of life.
